# Utilization of wheat germ oil and wheat bran fiber as fat replacer for the development of low‐fat beef patties

**DOI:** 10.1002/fsn3.1988

**Published:** 2021-01-22

**Authors:** Anam Khalid, Muhammad Sohaib, Muhammad Tahir Nadeem, Farhan Saeed, Ali Imran, Muhammad Imran, Muhammad Inam Afzal, Sana Ramzan, Muhammad Nadeem, Faqir Muhammad Anjum, Muhammad Sajid Arshad

**Affiliations:** ^1^ Department of Food Science Faculty of Life Sciences Government College University Faisalabad Pakistan; ^2^ Department of Food Science and Human Nutrition University of Veterinary and animal Sciences Lahore Pakistan; ^3^ Department of Diet and Nutritional Sciences University of Lahore Lahore Pakistan; ^4^ Department of Biosciences COMSATS University Islamabad Islamabad Pakistan; ^5^ Department of Food Science & Technology Government College University Faisalabad, Layyah campus, Faisalabad Pakistan; ^6^ Department of Environmental Sciences COMSATS University Islamabad Vehari Campus Pakistan; ^7^ Vice Chancellor Secretariat University of the Gambia Banjul The Gambia

**Keywords:** beef patties, wheat bran fiber and fat replacer, wheat germ oil

## Abstract

The present study was aimed to evaluate the effects of wheat germ oil and wheat bran fiber as fat replacers on quality and stability of low‐fat beef patties. Total five treatments were prepared by employing wheat germ oil (WGO) and wheat bran fiber (WBF). WBF was used at fixed amount of 3% in all treatments except control in conjunction with varying WGO concentrations as follows: 1.5%, 3%, and 4.5%. Prepared raw and cooked beef patties were stored at 4°C, and further analyses were carried out up to 21 days of storage period with intermittent evaluation interval of 7 days. Higher values of TBARS, peroxide, and cholesterol were observed in raw and cooked beef patties in control, whereas minimum values were found in treatment of beef patties prepared with WGO 4.5% + WBF 3%. The physicochemical parameters were observed by pH and hunter color values. pH was higher in cooked patties as compared to beef patties and showed increases with increase in WGO concentration and storage intervals. The sensorial attributes were observed which included different parameters, such as appearance, texture, taste, odor, and overall acceptability. Higher score was given by the panelists to control for both raw and cooked beef patties; however, minimum score for all sensory properties was found in group treated with WGO 4.5% + WBF 3% within acceptable limit. In nutshell, raw and cooked beef patties treated with WGO 4.5% plus WBF 3% showed better quality, stability, and reduced cholesterol content.

## Graphical abstract

In nutshell, raw and cooked beef patties treated with wheat germ oil 4.5% plus wheat bran fiber 3% showed better quality, stability, and reduced cholesterol content.

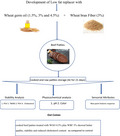

## 1 INTRODUCTION

Low‐fat meat products play very important role due to their health benefits and beneficial for all ages because of its nutritional and therapeutic value (Reyes‐Padilla et al., 2018). In recent era, foods that are low in fats and being in the daily meal of people have captured too much attention. By balanced proteins and daily diet levels, dietary fiber and its source levels should be included in balanced nutritional foods. Hence, due to high‐fat content, beef patties are now a best choice for fat reduction. Therefore, for the maintenance of healthy lifestyle, consumers demand those meat products having good quality as well as reduced fat (Ibarra Sáiz et al., 2012).

Nowadays, low‐fat meat products are used by the people who are conscious about their health. They considered these products for losing body weight because it is used as diet food. Meat continues to be the pivotal food in the whole world. By viewing the nutritional and sensory values of meat, the quality conditions are decided. The consciousness of the consumers on diet and health has expanded recently and the requirement for healthy foods, particularly meat which is a rich source of saturated fatty acids and monounsaturated fatty acids (Jalal et al., 2013).

There are different sources are used for the development of low‐fat meat products. Linseed and microalgal oils have been used as a fat replacer for the development of beef patties (Alejandre et al., 2016). However, there is a need to replace the fat contents with oils and fiber having functional as well as nutraceutical importance. In this regard, wheat germ oil and wheat bran fiber used to develop functional beef patties. Wheat germ oil (WGO) is the richest plant source of vitamin E and having great potential to lower down the fat contents (Arshad et al., 2013). Wheat germ comprises about 2.5%–3.5% of the total wheat and contained 10%–15% and phytosterols present which are helpful in lowering the fat contents (Arshad et al., 2013). In addition to wheat germ oil, wheat bran fiber was also used as a fat replacer. Higher contents of fiber present in bran and whole grain (Hemdane et al., 2016). The dietary guidelines for Americans recommend to utilize half of grains as whole grains to increase the intake of fiber which is necessary for excellent health (Jacobs et al., 2015). The major source of fiber is wheat bran which is used to cure constipation and diabetes and decreasing carcinogenic material. To reduce cancer, the activity of wheat is greater as compared to fiber content or increasing particular phyto‐chemical (Liu et al., 2012).

Half of the consumed food should contain whole grain. Less than 5% of Americans use the prescribed amount of whole grains which is equal to 3 ounce per day equivalent to whole grain. Frequently they consume less than 1 ounce equivalent to whole grain per day (Jonnalagadda et al., 2011). Keeping in view the above facts, the objective of the current study was to assess the impact of wheat germ oil and wheat bran fiber as fat replacers and to determine the effect of wheat germ oil and wheat bran fiber on quality and stability of raw and processed beef patties.

## 2 MATERIALS AND METHODS

This research project was carried out at Institute of Home and Food Sciences, Government College University Faisalabad, Pakistan. Wheat germ oil and wheat bran fiber were used as fat replacer for the development of functional beef patties and then further proceeded for chemical, physical analysis, and sensory attributes. Beef meat was obtained from local grocery stores from Faisalabad. Wheat germ and wheat bran were procured from Sunny flour mills Lahore, Pakistan.

### 2.1 Beef patties

Wheat germ oil and wheat grain fiber were used for the development of low‐fat beef patties by following the method. The meat patties were put away at 4**°**C for 21 days and investigation was done following seven days. At the point when all fixings were altogether blended, then a blend was exhausted in a slim layer (10 mm thickness) and shaped into round patties. In an expansive, nonstick griddle heat a large portion of the oil over medium‐low warmth and for around 4 min cook a large portion of the patties on each side or until brilliant dark colored. The preparation of beef patties with wheat bran fiber and different concentration of wheat germ oil is shown in Table 1.

**TABLE 1:** Preparation of beef patties with wheat bran fiber and different concentration of wheat germ oil

Ingredients (%)	Treatments				
	Control	WBF 3%	WGO 1.5% +WBF 3%	WGO 3% +WBF 3%	WGO 4.5% +WBF 3%
Lean meat	70	70	70	70	70
Back fat	10	10	10	10	10
Cold water	16.4	13.4	11.9	10.4	8.9
Salt	1.0	1.0	1.0	1.0	1.0
White pepper	0.2	0.2	0.2	0.2	0.2
Black pepper	0.2	0.2	0.2	0.2	0.2
Garlic powder	0.2	0.2	0.2	0.2	0.2
Onion powder	2.0	2.0	2.0	2.0	2.0
Wheat bran fiber	0.0	3	3	3	3
Wheat germ oil	0.0	0.0	1.5	3	4.5

### 2.2 Thiobarbituric acid reactive substances (TBARS) of beef patties

The TBARS value of raw and cooked beef patties was measured by following the method described by Ahn et al., 1998 with some modifications. The TBARS value was denoted by malondialdehydes/Kg of meat.

### 2.3 Peroxide value (POV) for beef patties

Peroxide value in raw and cooked beef patties was measured by method of International Dairy Federation (IDF) (Shantha & Decker, 1994). The POV was measured by using the unit mEq/Kg.

### 2.4 Hunter color value of beef patties

Utilizing a Hunter colorimeter with estimations institutionalized the surface shading estimation of the examples was performed regarding a white alignment plate (L = 89.2, a = 0.921, b = 0.783). The shading CIE L (lightness), CIE a (redness), and CIE b (yellowness) values were gotten utilizing a normal incentive from 9 arbitrary readings on each sample surface for statistical analysis.

### 2.5 Cholesterol contents

Total cholesterol content in raw and cooked beef patties was measured by using spectrophotometric method, as described by Rudel and Moris (1973). The cholesterol contents were measured by denoting the unit mg/100 g.

### 2.6 pH measurement

The pH of raw and cooked beef patties was estimated by utilizing pH meter as indicated by the method as depicted by AOAC, 1981.

### 2.7 Sensory evaluation

Sensory attributes of cooked beef patties were conducted by 10 panelists using 9‐point hedonic scale. The trained panelist was given mineral water to rinse their taste receptors for rational assessment. Different attributes like appearance, texture, odor, taste, and overall acceptability were assessed by the guidelines outlines by Lawless & Heymann, 2013.

### 2.8 Statistical analysis

Different parameters were analyzed statistically using a statistical software Statistics 8.1. Factorial design was used for measuring the level of significance by following the guidelines outlined by Steel and Torrie (2012). There were 10 measurements taken for hunter color determination and 10 panelists used for sensory evaluation. However, all the remaining data triplicates were taken.

## 3 RESULTS AND DISCUSSION

### 3.1 Thiobarbituric acid reactive substances (TBARS) value of raw and cooked beef patties

**FIGURE 1:**
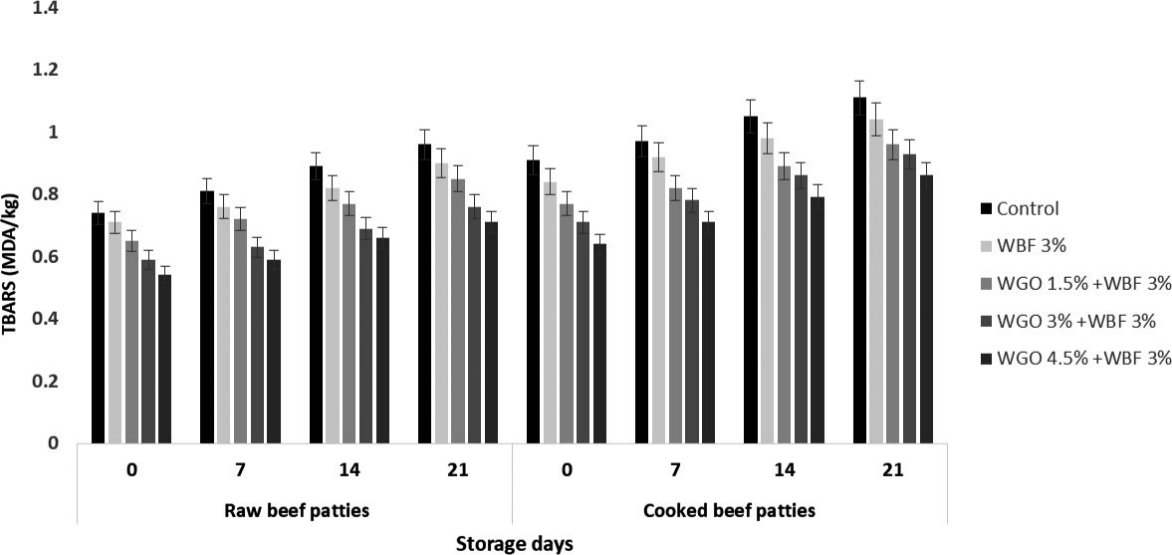
TBARS value of raw and cooked beef patties treated with wheat germ oil and wheat bran fiber during storage

The statistical results in regard to TBARS estimation of raw and cooked beef patties showed significant difference with respect to treatments and storage intervals as shown in Figure 1. Higher value of TBARS was observed in raw and cooked beef patties at day 21 of storage in control, whereas minimum value was found in raw and cooked beef patties treated with WGO (4.5%) and WBF (3%) at day 0 of storage. The outcomes showed that the TBARS value was higher in cooked in contrast with the raw beef patties and value increased with the passage of storage intervals which is in agreement with the findings of Arshad et al. (2017a). The results depicted that raw and cooked beef patties have minimum TBARS value where combination of WGO (4.5%) and WBF (3%) was used and is in line with the findings of Arshad et al. (2017b) where nuggets made from wheat germ oil have minimum TBARS value.

The results are in accordance with the outcomes of Rababah et al. (2006) who believed that cooking the samples significantly (*p* < .05) increased the amounts of TBARS and addition of plant extracts significantly decreased the amount of TBARS value. Furthermore, our results are supported by the findings of Kumar et al. (2016) who depicted that addition of wheat bran in chicken meat biscuits significantly reduced the TBARS value during storage period. Gómez et al. (2014) demonstrated that addition of grape seed extract and conjugated linoleic acid significantly reduced the TBARS value in the low‐fat beef patties.

### 3.2 Peroxide value of raw and cooked beef patties

Concentration of peroxides and hydroperoxides is measured by POV which is formed during auto‐oxidation of unsaturated fats in auto‐oxidation reaction (Jeon et al., 2002).

**FIGURE 2:**
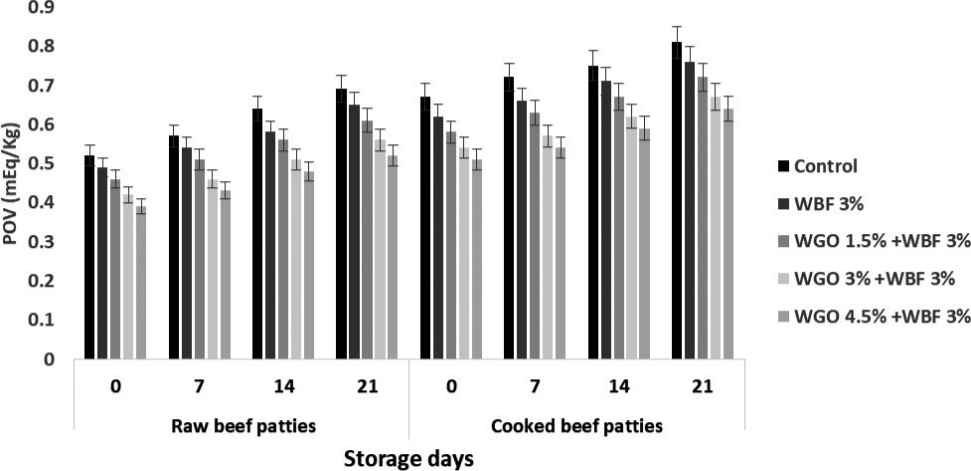
Peroxide value of raw and cooked beef patties treated with wheat germ oil and wheat bran fiber during storage

POV of raw and cooked beef patties has critical impact on treatments and storage intervals. Higher value of POV in raw and cooked beef patties in control was (0.69 ± 0.05 mEq/Kg), (0.81 ± 0.04 mEq/Kg) at 21 days of storage. Figure 2 presents that the minimum value of POV was found in raw and cooked beef patties treated with WGO (4.5%) and WBF (3%) was (0.39 ± 0.02 mEq/Kg) and (0.51 ± 0.04 mEq/Kg), respectively, at 0 day of storage. The results showed that the POV significantly decreased in raw beef patties samples as compared to cooked beef patties samples. Besides, the POV in cooked beef patties samples (in control condition) increased with the passage of time. The POV in raw and cooked beef patties samples also decreased with the addition of WGO (4.5%) and WBF (3%) in both samples. The results show that the raw beef patties have lower POV, while higher was found in cooked beef patties (in control condition) samples.

Harcourt et al. (2003) have detailed that unsaturated fats experience loss of hydrogen upon lipid peroxidation, bringing about the formation of a free radical at the site of unsaturation. At the point when the feedstock in which this reaction happens contains no vitamin E or other viable cancer prevention agent, the free radical is quickly changed over to a radical without unsaturated fat peroxide lastly into a hydroperoxide of the unsaturated fat. The diminished POV with expanded expansion of WGF might be because of nutrient E. (tocopherols) which are bottomless in wheat germ. Vitaglione et al. (2008) announced that germ establishes about 2.5% of grain weight and involves negligible measure of protein, yet most prominent offer of fat, nutrients particularly tocopherols. Winkler‐Moser et al. (2015) depicted that vitamin E is copious in wheat germ.

### 3.3 Cholesterol contents of raw and cooked beef patties

Cholesterol is expected to keep up typical cell work in people. About 70% of the required cholesterol is blended inside, while the staying 30% originates from creature nourishments, for example, eggs, meat items, and creature fats. However, cholesterol may experience oxidation to frame cholesterol oxidation (COP) items amid warming, presentation to light, or storage of cholesterol‐containing food items (Gopalakrishnan et al., 2016).

The statistical results regarding the cholesterol content of raw and cooked beef patties tests have critical impact concerning treatments and storage interval as shown in Table 2. Higher contents of cholesterol was found in control in both raw and cooked beef patties. Whereas Table 2 showed that minimum cholesterol contents was found in raw and cooked beef patties supplemented with WGO (4.5%) + WBF (3%). The results showed that the cholesterol level significantly was reducing in raw and cooked beef patties samples with the addition of wheat germ oil and wheat bran fiber.

**TABLE 2:** Cholesterol contents of raw and cooked beef patties treated with wheat germ oil and wheat bran fiber

Treatments	Raw beef patties	Cooked beef patties
Control	81.63 ± 3.15a	106.74 ± 3.58a
WBF 3%	79.65 ± 2.16b	104.39 ± 2.84b
WGO 1.5% + WBF 3%	76.74 ± 3.59c	99.64 ± 3.88c
WGO 3% + WBF 3%	72.36 ± 3.68d	96.38 ± 4.25d
WGO 4.5% + WBF 3%	71.48 ± 4.59e	92.18 ± 3.25e

Note

The values are mean ± *SD* of three independent determinations. Means carrying different letters in columns differed significantly.

WBF, Wheat bran fiber; WGO, wheat germ oil.

In concurrence with the result of Yildiz‐Turp and Serdaroglu (2008) who revealed that the meat containing pre‐emulsified sesame oil displacing pork fat had basically lower cholesterol substance (47.14–62.54 mg/100 g) differentiated and the control hitters which had 75.99 mg/100 g. Vegetable oils are free of cholesterol and have a higher extent of unsaturated to saturated fats than animal fats. It was also reported that lower cholesterol contents was observed in sucuks with pre‐emulsified hazelnut oil supplemented with 15, 30 and half of beef fat. Additionally, comparable outcomes were watched for matured sausage in various examinations exploring the impacts of supplanting pork fat/beef fat with pre‐emulsified olive oil (Kayaardi & Gök, 2004; Muguerza et al., 2001).

### 3.4 pH of raw and cooked beef patties

pH is a unit of measure the degree of acidity or alkalinity of a solution is described by pH on a scale of 0 to 14. The statistical results regarding the pH value of raw and cooked beef patties samples have significant effect with respect to treatments and storage interval as appeared Table 3. Higher value of pH was observed in raw beef patties in control at 21 day of storage and higher value of pH was observed in cooked beef patties samples at 21 day of storage, whereas Table 3 presents that the minimum value was found in raw beef patties samples 0 day of storage in control and minimum value was found in cooked beef patties sample in control at 0 day of storage. The results showed that the pH value significantly decreased in raw beef patties samples as compared to cooked beef patties samples. Besides, the pH value in cooked beef patties samples (control) increased with the passage of time. The pH value in raw and cooked beef patties samples also increased with the addition of WGO (4.5%) and WBF (3%) in both samples.

**TABLE 3:** pH value of raw and cooked beef patties treated with wheat germ oil and wheat bran fiber during storage

Treatments	Raw beef patties					Cooked beef patties				
	0	7	14	21	Mean	0	7	14	21	Mean
Control	6.02 ± 0.02	6.12 ± 0.15	6.19 ± 0.18	6.26 ± 0.16	6.15 ± 0.18d	6.34 ± 0.15	6.43 ± 0.16	6.52 ± 0.16	6.58 ± 0.06	6.47 ± 0.06c
WBF 3%	6.11 ± 0.06	6.19 ± 0.24	6.34 ± 0.16	6.37 ± 0.18	6.25 ± 0.05c	6.39 ± 0.18	6.46 ± 0.18	6.49 ± 0.16	6.53 ± 0.25	6.47 ± 0.16bc
WGO 1.5% + WBF 3%	6.15 ± 0.05	6.21 ± 0.03	6.29 ± 0.13	6.4 ± 0.19	6.26 ± 0.18b	6.42 ± 0.25	6.46 ± 0.18	6.52 ± 0.24	6.57 ± 0.23	6.49 ± 0.06b
WGO 3% + WBF 3%	6.13 ± 0.04	6.26 ± 0.16	6.31 ± 0.05	6.43 ± 0.17	6.28 ± 0.17b	6.38 ± 0.21	6.41 ± 0.25	6.46 ± 0.06	6.58 ± 0.18	6.46 ± 0.08b
WGO 4.5% + WBF 3%	6.18 ± 0.25	6.22 ± 0.17	6.35 ± 0.09	6.48 ± 0.07	6.31 ± 0.16a	6.45 ± 0.07	6.51 ± 0.21	6.52 ± 0.15	6.57 ± 0.03	6.51 ± 0.14a

Note

The values are mean ± *SD* of three independent determinations. Means carrying different letters in columns differed significantly.

WBF, Wheat bran fiber; WGO, wheat germ oil.

The increased pH may likewise be because of the increased TVBN in light of the corruption of nitrogenous substances.

Choi et al. (2008) showed that the addition of rice wheat fiber expanded the pH value. The pH of meat was higher in characterized patties with vegetable oil and rice wheat fiber than uncooked meat batter. The pH furthermore expanded when the meat batter was warmed in light of the fact that imidazolium, the essential R gathering of the amino corrosive histidine, was uncovered amid warming. So also López‐Vargas et al. (2014) found a pH decline in burgers with expansion of enthusiasm organic product albedo. The pH esteems diminished amid storage time in crude and cooked beef patties. The aggregation of acids created by lactic corrosive microscopic organisms instead of vigorous microorganism may have added to the drop in pH (Beriain et al., 2011). Hunter color value of raw and cooked beef patties.

### 3.5 L* (lightness) value of raw and cooked beef patties

Color is the most imperative quality parameter, which is seen by shoppers at first sight. Staining is considered as the quality imperfection and it might influence agreeableness of the item. The statistical outcomes in regard to the shading estimation of crude beef patties and cooked meat patties tests have noteworthy impact as for treatments and storage interval as appeared in Tables 4, 5, 6.

**TABLE 4:** L* value of raw and cooked beef patties treated with wheat germ oil and wheat bran fiber during storage

Treatments	Raw beef patties					Cooked beef patties				
	0	7	14	21	Mean	0	7	14	21	Mean
Control	52.54 ± 2.04	52.69 ± 2.36	53.69 ± 2.11	53.96 ± 1.37	53.22 ± 1.55c	54.69 ± 1.81	55.69 ± 1.46	56.89 ± 1.46	57.96 ± 1.19	56.31 ± 1.45c
WBF 3%	52.46 ± 1.08	52.85 ± 1.85	53.11 ± 1.55	53.69 ± 1.59	53.03 ± 1.45c	55.64 ± 1.15	56.75 ± 1.35	57.46 ± 1.16	58.96 ± 1.87	57.20 ± 1.36b
WGO 1.5% + WBF 3%	53.89 ± 1.81	54.36 ± 1.58	54.96 ± 1.38	55.49 ± 1.45	54.68 ± 1.26b	56.36 ± 1.26	57.46 ± 1.69	57.16 ± 2.19	58.46 ± 1.65	57.36 ± 1.37b
WGO 3% + WBF 3%	55.67 ± 1.36	56.96 ± 1.31	56.42 ± 1.24	57.89 ± 1.39	56.74 ± 1.15ab	58.49 ± 1.11	59.86 ± 1.15	60.12 ± 2.22	60.49 ± 1.36	59.74 ± 1.16ab
WGO 4.5% + WBF 3%	56.37 ± 1.22	56.89 ± 2.03	57.96 ± 1.29	58.99 ± 1.16	57.55 ± 1.45a	58.97 ± 1.69	59.49 ± 1.15	60.84 ± 2.12	61.28 ± 1.65	60.15 ± 1.77a

Note

The values are mean ± *SD* of ten independent determinations. Means carrying different letters in columns differed significantly.

WBF, Wheat bran fiber; WGO, wheat germ oil.

**TABLE 5:** a* value of raw and cooked beef patties treated with wheat germ oil and wheat bran fiber during storage

Treatments	Raw beef patties					Cooked beef patties				
	0	7	14	21	Mean	0	7	14	21	Mean
Control	11.45 ± 0.21	12.56 ± 0.11	12.11 ± 0.21	13.65 ± 0.12	12.44 ± 0.14c	13.57 ± 0.16	13.85 ± 0.18	14.59 ± 0.16	14.9 ± 0.16	14.23 ± 0.15bc
WBF 3%	11.96 ± 0.24	12.56 ± 0.14	13.25 ± 0.18	13.59 ± 0.08	12.84 ± 0.12c	13.25 ± 0.15	13.49 ± 0.21	13.96 ± 0.13	14.58 ± 0.11	13.82 ± 0.11c
WGO 1.5% + WBF 3%	12.48 ± 0.16	12.86 ± 0.16	13.49 ± 0.25	13.96 ± 0.09	13.20 ± 0.07b	14.78 ± 0.09	15.12 ± 0.23	15.69 ± 0.22	16.45 ± 0.21	15.51 ± 0.24b
WGO 3% + WBF 3%	12.65 ± 0.25	12.69 ± 0.18	12.88 ± 0.11	13.49 ± 0.11	12.93 ± 0.06ab	15.49 ± 0.12	16.48 ± 0.14	16.89 ± 0.21	17.11 ± 0.12	16.49 ± 0.13a
WGO 4.5% + WBF 3%	13.98 ± 0.14	14.52 ± 0.24	14.7 ± 0.14	14.79 ± 0.09	14.50 ± 0.11a	15.85 ± 0.04	16.74 ± 0.08	17.52 ± 0.16	17.89 ± 0.08	17.00 ± 0.11a

Note

The values are mean ± *SD* of ten independent determinations. Means carrying different letters in columns differed significantly.

WBF, Wheat bran fiber; WGO, wheat germ oil.

**TABLE 6:** b* value of raw and cooked beef patties treated with wheat germ oil and wheat bran fiber during storage

Treatments	Raw beef patties					Cooked beef patties				
	0	7	14	21	Mean	0	7	14	21	Mean
Control	8.69 ± 0.11	9.46 ± 0.21	9.79 ± 0.22	10.44 ± 0.15	9.60 ± 0.18b	9.78 ± 0.18	10.39 ± 0.21	10.34 ± 0.21	10.25 ± 0.11	10.19 ± 0.11c
WBF 3%	9.68 ± 0.08	10.45 ± 0.25	10.36 ± 0.13	10.63 ± 0.16	10.28 ± 0.13ab	10.56 ± 0.16	11.36 ± 0.14	11.26 ± 0.12	11.96 ± 0.14	11.29 ± 0.12b
WGO 1.5% + WBF 3%	9.45 ± 0.14	9.51 ± 0.21	9.65 ± 0.15	10.49 ± 0.12	9.78 ± 0.11b	10.82 ± 0.16	11.36 ± 0.18	11.96 ± 0.19	12.03 ± 0.06	11.54 ± 0.11b
WGO 3% + WBF 3%	9.96 ± 0.26	10.45 ± 0.11	10.77 ± 0.18	10.69 ± 0.19	10.47 ± 0.16ab	10.56 ± 0.14	10.39 ± 0.16	11.63 ± 0.08	11.03 ± 0.19	10.90 ± 0.13c
WGO 4.5% + WBF 3%	10.36 ± 0.11	11.36 ± 0.15	11.52 ± 0.11	11.82 ± 0.22	11.27 ± 0.15a	11.96 ± 0.12	12.63 ± 0.12	12.96 ± 0.11	12.05 ± 0.04	12.40 ± 0.11a

Note

The values are mean ± *SD* of ten independent determinations. Means carrying different letters in columns differed significantly.

WBF, Wheat bran fiber; WGO, wheat germ oil.

Higher value of L* was observed in raw beef patties at day 21 day with addition of WGO (4.5%) and WBF (3%) and in cooked beef patties samples (61.28 ± 0.05) at day 21 with addition of WGO (4.5%) and WBF (3%), whereas Table 4 presents that the minimum value (52.46 ± 0.01) was found in raw beef patties samples at 0 day of storage with addition of WBF (3%) and minimum value was found in cooked beef patties sample in control at 0 day of storage. The results showed that the L* value significantly increased in cooked beef patties samples as compared to raw beef patties samples. Besides, the L* value in raw beef patties samples (control) increased with the passage of time. The L* value in raw and cooked beef patties samples also increased with the addition of WGO (4.5%) and WBF (3%) in both samples.

Zembayashi et al. (1999) affirmed that there was a connection (*y* = 64.37–19.35*x* + 3.54*x*2, *r*2 = 0.79) between iron focus and L* esteem (lightness), and recommended that meat color could be improved by a decrease in iron centralization of muscle. The results acquired were in concurrence with the examination finding of (Salvador & Fiszman, 2004) who revealed that no critical change in color was found amid the storage. The incomplete substitution of meat fat by olive and linseed oils' blend added to a fundamentally higher L ∗ values. A few creators have announced that fat alteration by oil is related to high daintiness of the completed item (Beriain et al., 2011; Martinez‐Finkelshtein et al., 2012).

### 3.6 a* (redness) value of raw and cooked beef patties

Higher value of a* in raw and cooked beef patties was observed at 21 days of storage with addition of WGO (4.5%) and WBF (3%), whereas Table 5 presents that the minimum value (11.45 ± 0.42) was found in raw beef patties samples (control) at 0 day, and in cooked beef patties, the minimum value was observed in treatment having WBF (3%) at 0 day of storage. The results showed that the a* value significantly decreased in raw beef patties samples as compared to cooked beef patties samples. Besides, the a* value in cooked beef patties samples increased with the passage of time. The a* value in raw and cooked beef patties samples also increased with the addition of WGO (4.5%) and WBF (3%) in both samples. The results showed that the raw beef patties have lower values of a*, while higher was found in cooked beef patties (control) samples.

Gili et al. (2017) revealed that the oil quality parameters showed that the raw germ had a period length of convenience of around 15 days, with the warmth treated wheat germ keeping up its quality for no under 90 days under these stored conditions. The color is a pointer of procedure quality control as brown colors increment as the tanning and caramelization responses advance. In this way, the control of color changes is by all accounts important to get good product quality. ± while a * and b * were higher. An increase in saltiness diminished (*p* < .01) redness (a *) of patties. Redness values of uncooked and cooked meat hatters were lower in batters formulated with vegetable oils. Comparative results were accounted for by Paneras and Bloukas (1994) for vegetable oils substituted for pork back fat in low‐fat frankfurters and by Park and Park (2005) in regard to the quality properties of low‐fat burger patties with included plant oils. The most reduced yellowness values were found in the control uncooked and cooked meat batters.

### 3.7 b*(yellowish) value of raw and cooked beef patties

Higher value of b* in raw and cooked beef patties was observed at day 21 and at day 14 (11.82 ± 0.31) and (12.96 ± 0.33), respectively, with addition of WGO (4.5%) and WBF (3%), whereas Table 6 presents that the minimum value of raw and cooked beef patties was found and in control at 0 day of storage. The results showed that the b* value significantly decreased in raw beef patties samples as compared to cooked beef patties samples. Besides, the b* value in cooked beef patties samples in control increased with the passage of time. The b* value in raw beef patties and cooked beef patties samples also increased with the addition of WGO(4.5%) and WBF (3%)in both samples. The results show that the raw beef patties have lower values of b*, while higher was found in cooked beef patties in control samples.

Saricoban and Yilmaz (2010) reported that the increase in b * values may be due to a decrease in the content of oxymyoglobin, the major pigment responsible for the yellow color in meat products. Decreasing the b * value indicates that the meatball's color has turned blue rather than yellow. The decrease in yellow color could be due to the decrease in oxymyoglobin content. It has been reported that the index b *, which is inversely proportional to the oxymyoglobin content, decreases at the beginning of the salting of the meat products Choi et al., (2012) studied the effects of rice bran on sensory and physicochemical properties of emulsified pork meatballs and reported that addition of wheat dietary fiber increased the b* value.

### 3.8 Appearance and texture of cooked beef patties

Appearance and texture are an important quality parameter in different food commodities. Improper texture and appearance do not attract the attention of the consumers.

**FIGURE 3:**
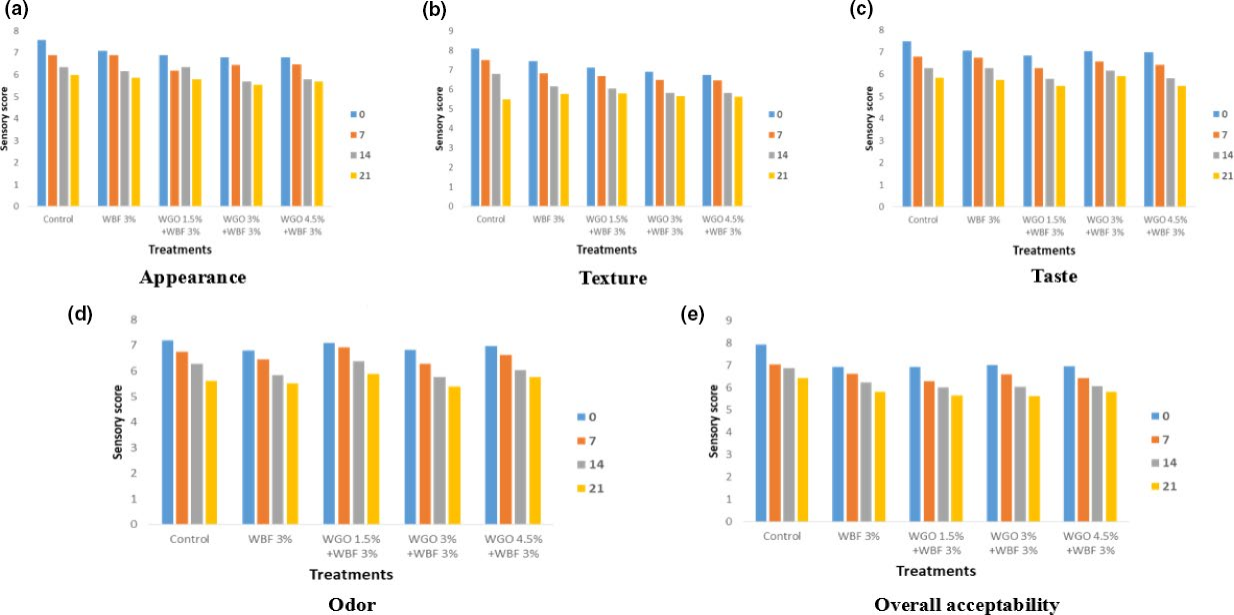
(a) Appearance of cooked beef patties during storage, (b) texture of cooked beef patties during storage, (c) taste of cooked beef patties during storage, (d) odor of cooked beef patties during storage, (e) overall acceptability of cooked beef patties during storage

The statistical results regarding the appearance and texture value of cooked beef patties samples have significant effect with respect to treatments and storage interval as shown in Figure 3.

Higher value of appearance and texture was observed in control at 0 day of storage, whereas Figure 3 presents that the minimum value of appearance and texture was found in cooked beef patties treated with WGO (3%) and WBF (3%). The results showed that the appearance and textures score significantly decreased in cooked beef patties samples. The appearance and texture score in cooked beef patties samples also decreased with the addition of WGO (4.5%) and WBF (3%) in both samples. The results showed that the score for appearance and texture decreased with the passage of time or with the addition of WGO and WBF.

The results were in concurrence with Mumtaz et al. (2008), who announced in his examination discoveries that surface was influenced fundamentally amid capacity. It was bolstered by Herrero and Requena (2006), who found that the textural properties continued as before all through the storage period. Chewiness values significantly increased by fat replacers percentages increment. Also sausage prepared with hydrated wheat bran had significantly higher chewiness values than that prepared with hydrated barley whole meal. Springiness value for all sausage treatments ranged from 0.60 to 0.75 showed no significant differences between all treatments. These outcomes were in accordance with the outcomes detailed by certain specialists on various cooked sausages (Herrero et al., 2008). Our outcomes likewise concur with Ayadi et al. (2009) who detailed that κ/ι‐carrageenan expansion to turkey sausages expanded hardness and water restricting limit.

### 3.9 Taste and Odor of cooked beef patties

Taste is also an important sensory characteristic. On the tongue, it is recognized by the taste buds and odor is also very important sensory characteristic. The statistical results regarding the taste and odor value of cooked beef patties samples have significant effect with respect to treatments and storage interval as shown in Figure 3. Higher value of taste and odor was observed in control at 0 day of storage, whereas Figure 3 presents that the minimum value of taste of beef patties was found which is treated with WGO (3%) and WBF (3%) at 21 day of storage and minimum value of odor was found in cooked beef patties sample at 21 days of storage. The results showed that the texture and odor value significantly decreased in cooked beef patties samples. The taste and value of cooked beef patties samples also decreased with the addition of WGO(4.5%) and WBF (3%)in both samples. The results showed that the value of taste and appearance decreased with the passage of time or with the addition of WGO and WBF.

Beef patties formulated with wheat bran that had no adverse effects on the acceptability of their appearance, color, and their odor. Moreover, the formulation of beef patties with partial replacement of different cereal bran produced acceptable samples compared with the control in their good flavor, sufficient juiciness, consistent texture (Feiner, 2006). It has been reported that wheat fiber is neutral in taste and helps moisture and fat to produce more stable and juicy meat products (Ramadan et al., 2016).

### 3.10 Overall acceptability of cooked beef patties

The overall acceptability of the cooked beef patties is also a quality index of the product. The results regarding analysis of variance for overall acceptability of the prepared patties samples are presented in Figure 3. The statistical results regarding the overall acceptability value of cooked beef patties samples have significant effect with respect to treatments and storage interval as shown in Figure 3. The higher values of acceptability showed at 0 day of storage in control. Minimum values observed in cooked beef patties at 21 day in samples which treated with WGO 4.5% +WBF 3% that depicted 5.83 ± 0.21. The trend showed the gradual decrease of scores from control to the treated samples. Similar trend was depicted by the storage days that showed lower values of taste from 0 to 21 days. However, a different behavior and trend were measured at treatment level WGO 3% + WBF 3% that deviate from general trends showed sudden increase of accessibility values at each storage day. The mean values of cooked beef patties gradually decrease at each treatment level.

The slight increment away misfortune with increment of storage period that might be because of dissipation of dampness and this concurs with the finding of Varnam et al. (1995), who detailed that for the most part the outcome showed that dampness substance of all examples diminished after storage because of vanishing misfortune ordinarily saw amid storage at two distinctive temperature, similar to the misfortune happens as aftereffect of vanishing of the water from meat surface, when brought from a virus store into conventional room temperature. An increase in the storage loss leads to decrease in moisture content. Other authors have reported no significant differences in sensory properties (odor, color, taste, hardness, juiciness, and fattiness) for other fresh meat products (burger patties) reformulated with a polyunsaturated gelled emulsion as a replacer for pork back fat replacer (Poyato et al., 2015).

## 4 CONCLUSIONS

This study was designed to explain the influence of wheat germ oil and wheat bran fiber as fat replacers on quality and stability of low‐fat beef patties. TBARS and POV for raw and cooked beef patties were found to be higher in control whereas higher value was observed in group having higher concentration of WGO and WBF. Similar trend was also observed for the cholesterol contents. pH was higher in cooked patties as compared to beef patties and showed increases with increase in WGO concentration and storage intervals. Higher score was given by the panelists to control for both raw and cooked beef patties; however, minimum score for all sensory properties was found in group treated with WGO 4.5% + WBF 3% within acceptable limit. In nutshell, raw and cooked beef patties treated with WGO 4.5% plus WBF 3% showed better quality, stability, and reduced cholesterol content.
